# Feasibility of Non-Sedate Magnetic Resonance Imaging for Children with Cerebral Palsy: Tolerance and Structural Analysis Considerations

**DOI:** 10.3390/children13040560

**Published:** 2026-04-17

**Authors:** Stefanie S. Bradley, Elizabeth Pulcine, F. Virginia Wright, Manohar Shroff, Kevin Chung, Tom Chau

**Affiliations:** 1 Bloorview Research Institute, Holland Bloorview Kids Rehabilitation Hospital, Toronto, ON M4G 1R8, Canada; stefanie.bradley@mail.utoronto.ca (S.S.B.); vwright@hollandbloorview.ca (F.V.W.); kchung@hollandbloorview.ca (K.C.); 2 Institute of Biomedical Engineering, University of Toronto, Toronto, ON M5S 1A1, Canada; 3 Division of Neurology, The Hospital for Sick Children, Toronto, ON M5G 1R8, Canada; liza.pulcine@sickkids.ca; 4 Department of Paediatrics, University of Toronto, Toronto, ON M5S 1A1, Canada; 5 Department of Physical Therapy, University of Toronto, Toronto, ON M5S 1A1, Canada; 6 Department of Diagnostic and Interventional Radiology, The Hospital for Sick Children, Toronto, ON M5G 1R8, Canada; manohar.shroff@sickkids.ca; 7 Department of Medical Imaging, University of Toronto, Toronto, ON M5S 1A1, Canada

**Keywords:** cerebral palsy, pediatric, neuroimaging, MRI, GMFCS IV, Freesurfer, structural preprocessing, motion, feasibility

## Abstract

**Highlights:**

**What are the main findings?**

• Primary school-aged children with cerebral palsy (CP) had a ~50% completion rate for structural brain MRI without sedation, and tolerance for awake MRI correlated with gross motor ability.
• In a proof-of-concept motion analysis, Freesurfer segmentation of the basal ganglia, compared with manual brain segmentation, showed that motion least affected the caudate and most affected the pallidum.

**What are the implications of the main findings?**

• Despite advances in automated MRI processing tools, motion-affected pediatric scans require manual correction after software auto-segmentation.
• Additional preparation and tailored accommodation are needed for children with CP and their families during non-sedate MRIs to promote inclusion of underrepresented populations in research studies.

**Abstract:**

**Background/Objectives**: Non-sedate magnetic resonance imaging (MRI) can be challenging for young children with neuromotor disabilities, often resulting in motion-degraded images that complicate interpretation in the context of underlying neuropathology. This study aimed to characterize tolerance factors and barriers related to awake MRI of the pediatric brain and to examine additional considerations in analyzing structural scans affected by motion and pathology. **Methods**: 10 children (mean age 5y9m; 5 girls; GMFCS level IV) with cerebral palsy (CP) underwent non-sedate 3T MRI of the brain. Tolerance factors and challenges were documented. MRI quality and automated structural preprocessing with Freesurfer (FS) v.8.0 were reviewed by a pediatric neuroradiologist and neurologist. To assess the impact of motion, automated basal ganglia segmentation was compared with manual segmentation. Segmentation accuracy was characterized using Dice Coefficient (D). **Results**: Five participants (50%) tolerated non-sedate structural MRI, although two of them were unable to remain still. Factors affecting MRI tolerance included sensitivity to scanner noise (*n* = 4), hyperkinetic movement (*n* = 2), difficulty with positioning/padding (*n* = 4), fear of clinical environment (*n* = 2) or confined scanner interior (*n* = 2), and earbud discomfort (*n* = 3). Automated structural preprocessing with FS yielded discrepancies in gray-white matter boundaries in motion-degraded scans, necessitating manual correction. Automated segmentation of motion-compromised scans closely agreed with manual delineation of the caudate (D ≥ 0.85) and putamen (D ≥ 0.78), while the pallidum was least reproducible (D = 0.58). **Conclusions**: Tailored acquisition and processing strategies are necessary to support non-sedate MRI in children with CP, preserve downstream neuroimaging analyses, and promote inclusion of underrepresented populations in research.

## 1. Introduction

Children with cerebral palsy (CP) or acquired brain injury undergo clinical neuroimaging as an important step in their diagnostic work-up and prognostic considerations [1]. These early-age clinical neuroimaging scans often occur as sleep scans (<2 years old) or with sedation. In contrast to diagnostic imaging, magnetic resonance imaging (MRI) in elective pediatric research studies is typically conducted without sedation, due to the high cost of anesthesia and potential safety risks associated with anesthetic medications [2]. Thus, research studies with preschool-aged children that include neuroimaging may be a child’s first exposure to an awake brain MRI. Families may view optional research MRIs for their child with greater reservation than MRI integral to clinical care [3]. For children with neurodevelopmental disabilities, this reluctance is heightened by concerns about sensory overstimulation and the need to remain motionless in a constrained environment [4].

Tolerability for awake neuroimaging in children with neurodevelopmental conditions (ADHD, ASD) is lower (70–81% success rate) than neurotypical controls (~87%) [5], even when using a mock scanner for practice beforehand [6]. Most successful non-sedate neuroimaging studies for children with CP have been with those who are independently ambulatory, i.e., those categorized as Gross Motor Function Classification System Level I (GMFCS I) [7,8]. In comparison, there is a lack of literature regarding factors relating to successful acquisition of non-sedate brain MRIs in minimally or non-ambulatory children functioning at GMFCS level IV or V. Children functioning at this motor level are more likely to have comorbidities that include behavioral difficulties, cognitive impairment, global developmental delays, varying expressive and receptive communication difficulties, epilepsy, auditory and/or visual impairments, and a range of orthopedic abnormalities [9]. In addition, children with CP are more likely to have increased startle response and involuntary movements like dystonia that can cause motion artifacts in the acquired images [10]. Though children with CP may be prescribed mild sedatives for muscle spasticity management in the context of clinical care, research hospitals often do not have the infrastructure supports and medical monitoring capacity to support higher-risk research protocols for MRIs, especially in the case of medically complex children where the theoretical use of oral sedatives for a research MRI scan may pose risk for respiratory depression [11].

Structural estimates of white matter (WM) and gray matter (GM) volumes and cortical thickness in brain MRI are affected by motion artifacts in a dose-dependent manner [12]. In pediatrics, reliable segmentation is further complicated by smaller regions of interest, reduced GM/WM contrast depending on age-appropriate myelination, within-tissue intensity heterogeneities, and variable head circumference and shape relative to MRI head coils [13,14]. Though manual segmentation remains the gold standard for neuroanatomical delineation, it is often not feasible for large-cohort studies due to its labor-intensive and time-consuming nature. Thus, automated and semi-automated pipelines have become common for processing of neurotypical T1-weighted (T1W) scans [15]. For children with neurodevelopmental disabilities, additional considerations are necessary when using automated segmentation tools, as most algorithms are trained on adult data [13] without consideration for myelination patterns, varying brain anatomy or neuropathology.

For individuals with CP, structural brain abnormalities are present on approximately 80% of MRI scans [1]. Most common pathologies include (i) periventricular leukomalacia secondary to hypoxic–ischemic encephalopathy, (ii) posthemorrhagic hydrocephalus and secondary ex vacuole ventriculomegaly due to prematurity, and (iii) neonatal or presumed perinatal arterial ischemic stroke [16]. Involvement of the basal ganglia is commonly seen in those functioning at GMFCS IV or V [17]. Freesurfer (https://surfer.nmr.mgh.harvard.edu/accessed on 10 January 2023) is a common neuroimaging analysis software package that has been validated in children 5 years old and up when using appropriate quality assessment measures [18] and for non-neurotypical children (ASD, ADHD, epilepsy) [19,20]. It has not yet been validated for children with CP functioning at GMFCS IV.

Participation of a heterogenous pediatric population in neurodevelopmental research studies of brain integrity and functioning is important both for generalizability of study results and inclusive and patient-centered, real-world applicability. Brain characterization at various stages of development can reveal associations with clinical presentations and functionality [21], as well as rehabilitation efficacy of interventions [22]. Not surprisingly, children in GMFCS IV and V are underrepresented in research studies [23] and addressing this gap can reduce sampling bias toward neurotypical children [24] as well as promote the neuromechanistic understanding of early intervention or therapies in the young developing brain [25]. However, the most neurologically involved children are prone to the highest motion artifacts in an MRI, raising concerns about accuracy and interpretability of results. Thus, it is necessary to understand the capabilities of automated neural segmentation tools when analyzing structural brain MRIs in children with neurodevelopmental disabilities of varying severity to ensure that the resulting segmentations are sufficiently accurate for drawing valid study conclusions.

To our knowledge, there are no published studies investigating the feasibility of non-sedate neuroimaging in children functioning within GMFCS IV. The objective of our research study was to investigate the success rate (including relevant factors and barriers) of completing structural brain MRIs within a multi-method research protocol among a representative sample of young children with CP (GMFCS IV). We aimed to report structural brain characterization, motion quality assessment, and considerations for structural analysis using a semi-automated Freesurfer software pipeline for gray matter (GM) and white matter (WM) delineation, with basal ganglia segmentation included as a representative subcortical structure of relevance in CP.

## 2. Materials and Methods

### 2.1. Participants

This study explored the MRI experience and baseline structural scans (T1W) of 10 participants (5 girls; mean age 5y9m. [4y3m.–5y3m.]; 6/10 dyskinetic CP; 3/10 spastic CP, 1/10 genetic syndrome clinically presenting with ataxic CP) who were participants in a physiotherapy feasibility study approved by the research ethics committee of Holland Bloorview Kids Rehabilitation Hospital (no. 0523), and the University of Toronto (no. 00044118), in accordance with the Tri-Council Policy Statement in Canada [26].

Participants were recruited from their circle of care (pediatricians and physiotherapists) and met the following inclusion criteria: (i) aged 3–6 years inclusive at the time of study invitation; (ii) CP classified as GMFCS IV or equivalent: uses a wheelchair (pushed by others or powered) most of the time, and walking is very limited even with use of assistive devices, and (iii) fulfilled MRI eligibility requirements. Other inclusion and exclusion criteria are detailed in the corresponding study protocol paper [26].

Neuroimaging consent was obtained as part of the overarching research study consent, with an additional MRI requisition for each participant reviewed by the MRI technologist (K.C.) prior to scheduling the MRI scans. Completed scans were reviewed by a pediatric neuroradiologist (M.S.) for any incidental findings that would be communicated to the study pediatrician.

As an age-matched reference, a NIH pediatric anatomical T1W template (natural asymmetric; mean of children aged 4.5–8.5 years old) [27] was included in this study to represent a neurotypical (NT) control scan without presence of motion artifacts or pathology.

### 2.2. Study Visits and MRI Preparation

As part of the physiotherapy study, each family had a total of 17 study sessions (in-person screening (1 visit), physical assessments (3 visits) and 13 intervention-related sessions) apart from the MRI visits included in this study. The intensity of this protocol provided an opportunity to examine how families of children view and prioritize research MRI participation within the context of a busy, multi-visit study. If tolerated, MRI scans were done at study baseline and immediately post-intervention, with an option for a third scan at 1-month post-intervention. If a child could not tolerate the baseline scan, they were not asked to return for subsequent MRI timepoints.

Prior to the baseline scan, families were given MRI resources intended to prepare and educate them for an awake scan with their child [28]. Resources included a child-friendly MRI explanation book, video links to a tour of the center’s MRI suite, links to MRI cartoons on YouTube and MRI sound samples, and a link to a resting state fMRI video. This was intended to ease any apprehension or stress associated with the MRI process. If time permitted, on the day of the scan there would be a game provided by S.S.B. (such as a scavenger hunt) that led down to the MRI suite.

### 2.3. MRI Image Acquisition

**Figure 1:**
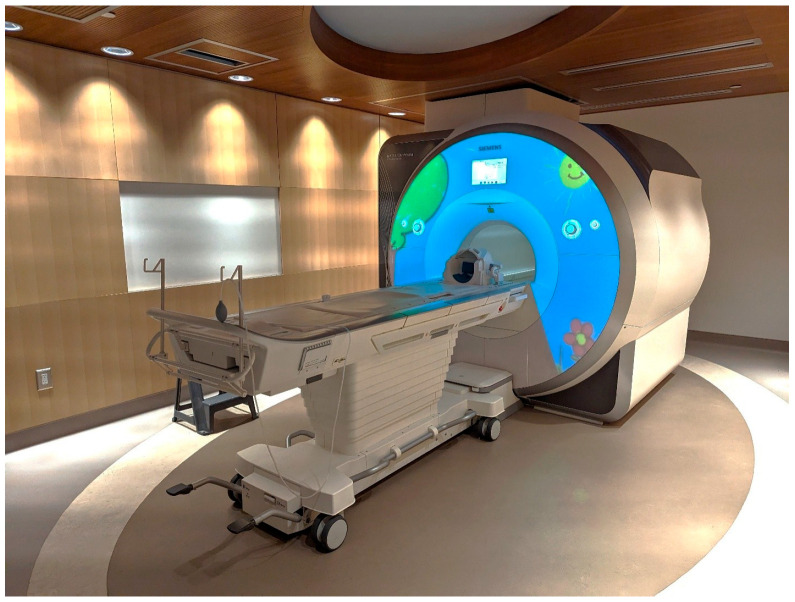
MRI suite at Holland Bloorview Kids Rehabilitation Hospital. This suite is fully accessible, child-friendly, research-focused, immersive, and customizable. Child-friendly elements of the MRI suite include child-friendly projections on the magnet bore, removable MRI scanning bed to accommodate transfers and acclimation prior to enter the scan room, customizable wall and ceiling projections, dimmable and color adjustable comfort lighting, smart glass door, and MRI-compatible audiovisual goggles (not shown). Non-sedate structural MRI of the brain was performed pre- and post-intervention, with the option of a third follow-up MRI.

Awake MRI head scanning was done in a Siemens Prisma MAGNETOM 3-Tesla MRI scanner (Siemens Healthcare, Erlangen, Germany) with a 32-channel head coil at Holland Bloorview Kids Rehabilitation Hospital (Figure 1). Scanning protocol parameters were selected with the guidance of an MRI technologist (K.C.) to prioritize a short acquisition time (31:38 min duration) for the young demographic. MRI scan protocol included T1W scanning, T2W scanning, diffusion kurtosis imaging (DKI), and resting state functional MRI (rs-fMRI). For the T1W scan (3D MPRAGE; 5:03 min duration), the voxel size was 0.8 mm isotropic, FOV = 256 mm3, TR = 3.1 ms and TE = 1870 ms for 240 sagittal slices.

Following review of the MRI safety screening forms of the caregiver and child, the MRI scan procedure was explained by the technologist. The child was changed into MRI-safe clothing next. To provide the option to alleviate anxiety and optimize comfort, the MRI table was detached and moved out of the MRI scan room to set up some of the participants. For those who could be set up inside the MRI scan room, an MRI compatible wheelchair was available. Parents also had the option to carry their child into the scan room to be placed on the scanner table. Holland Bloorview’s MRI suite is fully accessible and features adjustable lighting with child-friendly projections that could be customized for each child. Participants were also permitted to bring a metal-free stuffed animal or a blanket from home to have in the scanner. Pillows and padding were placed around the child to ensure appropriate scan positioning, reduce head motion and provide comfort. Earplugs or earbuds were inserted prior to the anterior head coil helmet. During this stage, the caregivers would often play a game like finger puppets for distraction. Parents could opt to remain in the scan room or partially lie in the scanner with their child during the exam to improve participant acceptance. If significant motion occurred causing artifacts on the acquired images, the scan sequence would be repeated at the discretion of the MRI technologist (K.C.) and study researcher (S.S.B.) while taking into consideration the participant’s tolerance of extra time in the magnet’s bore.

While inside the scanner, all children were monitored closely by their caregiver, the MRI technologist, and study researcher. Participants wore noise-reducing headphones and watched videos of their choice during structural scans, and the visual paradigm Inscapes video during fMRI scanning. The children were given an emergency call button and scans were stopped if there was any significant distress from the child or signals from their caregiver accompanying them in the scan room.

### 2.4. Tolerability Metrics

Tolerance in this study was defined as procedural adherence to the MRI scanning protocol without significant stress or other adverse reactions of the child. Factors related to tolerability such as extent of MRI scan completion, observed MRI session challenges, and family feedback were tracked on a log sheet by the study researcher (S.S.B.) for each child. These MRI session observations were recorded as qualitative notes to capture behavioral responses so that MRI challenges could be categorized and quantified through frequency counts.

To explore associations with a child’s MRI tolerance, participant characteristics were drawn from the baseline physiotherapy assessments of the study [29] which included the Gross Motor Function Measure (GMFM) Lie and Sit domains [30], Manual Ability Classification System (MACS) [31], Communication Function Classification System (CFCS) [32], and Parent-reported Dimensions of Mastery Questionnaire (DQM) [33]. The child’s CP subtype was provided by the study pediatrician according to the SCPE classification of CP [34].

### 2.5. Scan Quality and Characterization

Completed MRI scans were reviewed by a pediatric neurologist (E.P.) and pediatric neuroradiologist (M.S.) to assess scan quality and the presence of any pathology. Radiological findings in cortical or subcortical areas were documented to characterize the brains of children who could tolerate the MRI.

Image quality assessment based on the overall appearance of the entire native T1W volumes considered image sharpness, ringing, and contrast-to-noise ratio (CNR) for GM, WM, and subcortical structures, as per Backhausen et al. [12]. A four-point rating scale derived from systematic pediatric parameters noted in White et al. [35] was used—Excellent (crystal clear); Fair (some blurring); Poor (significant blurring but some differentiation); Unusable (no differentiation). All scans were qualitatively described in terms of radiologic findings, but anything rated as “Unusable” was not retained for further structural preprocessing.

Scan quality assessment (QA) was conducted post-Freesurfer processing with the quantitative tool qatools.py (https://github.com/Deep-MI/qatools-python accessed on 1 September 2023), a script which generates a set of MRI quality metrics such as CNR, signal-to-noise ratio (SNR), and number of defects (topological errors, atypical anatomy, or geometric abnormalities) in the reconstructed surface.

### 2.6. Processing of T1W Images

T1W structural images were processed using the automated cross-sectional reconstruction processing stream (recon-all) from Freesurfer (FS) version 8.0.0 on a Linux Ubuntu system version 22.04.3. Briefly, these steps included motion correction, intensity inhomogeneity correction, bias field correction, skull stripping, GM/WM boundary tessellation, topology correction, registration to a spherical atlas, and cortical reconstruction and automated brain structure labeling [36]. Freesurfer v.8.0 contains deep learning integration that was not present in previous versions: SynthSeg, a convolutional neural network that is more robust to variable scan contrasts or resolutions [37] and SynthStrip for improved skull stripping on pediatric scans [38]. Any scans that had a subsequent timepoint available were also subjected to the FS longitudinal processing pipeline, a within-child unbiased template used to reduce noise [39], as an additional reliability measure [40]. Scans that remained substantively compromised by motion artifacts (visible ghosting or blurring, striping across slices, distorted brain boundaries) and were unable to be processed by the recon-all pipeline were omitted from subsequent structural segmentation steps. The NIH pediatric anatomical T1W template was also processed through recon-all.

### 2.7. Manual Editing and Segmentations

Cortical GM/WM delineations made with FS were visually inspected by a pediatric neuroradiologist (M.S.). If GM/WM boundaries were deemed inaccurate, these reconstructed scans were manually edited by modifying the white matter mask, eliminating non-brain tissue voxels, or using control points for intensity normalization errors, using the semiautomated pediatric segmentation protocol [18] and FS’s documentation (“Recon Editing Guidelines for Child MRI”) as a guideline. Once edits were complete, the recon-all pipeline was re-run and the updated cortical GM/WM delineations were verified by the pediatric neuroradiologist (M.S.) to ensure accuracy.

FS’s automated subcortical structure segmentations (aseg) are derived from probabilistic location information based on Bayesian inference, from a manually labeled training set [41]. As a representative subcortical brain structure relevant to individuals with CP [17], segmentations of basal ganglia components (caudate, putamen, and globus pallidus) were chosen to compare accuracy with those that were manually segmented as ground truth.

Manual segmentations of the basal ganglia were done by study researcher (S.S.B.) using MINC Toolkit for MNI Display software version 1.9.18 (Montreal Neurological Institute, McGill University) to delineate the caudate, putamen, and globus pallidus. Tracings were done on successive slices in an axial view and then edited in the other orthogonal views, following the basal ganglia anatomical boundaries demonstrated in the IMAOIS platform (www.imaios.com accessed on 23 May 2025), and Alkemade et al. [42]. All manual segmentations were verified by a pediatric neuroradiologist (M.S.). If deemed inaccurate, manual segmentations were corrected.

### 2.8. Analyses

Tolerability metrics were quantified as frequency counts admitting either the entire study sample (*n* = 10) or the subset who tolerated at minimum the structural T1W scan (*n* = 5). Each child’s MRI tolerance was summarized as a binary yes/no and then tests for correlation with known participant characteristics (measurement scales as ordinal variables) were conducted with point-biserial correlation coefficients (r_pb), with *p* < 0.05 as statistically significant. Tests for association with CP subtype (for *n* = 9; genetic syndrome excluded) as a categorical variable was done using Fisher’s exact test (*p* < 0.05 as statistically significant) and an odds ratio (yes/no tolerance of dyskinetic participants over yes/no tolerance of participants). Challenges associated with the MRI experience were documented by a study researcher (S.S.B.), and a directed content analysis approach [43] taken to categorize and summarize via frequency counts for all 10 children, with multiple categories possible for each child.

For segmentation accuracy of the basal ganglia as a proof of concept, the percent volume difference (PVD) was used to quantify any volumetric differences between automated and manual segmentations of structures for each participant [44]:

(1) $$PVD=100\times \frac{\left|V_{A}-V_{M}\right|}{\left(V_{A}+V_{M}\right)/2}$$

where V_A_ and V_M_ are volumes of the structure of interest due to automatic and manual segmentation, respectively. The Dice coefficient (D) [40] was used to calculate structural similarity (spatial overlap) between the voxels in the manual and automated segmentations (value between 0.0 and 1.0; 1.0 = perfect spatial overlap).

(2) $$D=100\times \frac{\left|A\cap M\right|}{\left(\left|A\right|+\left|M\right|\right)/2}$$

where A and M are the set of voxels labeled as the structure of interest by automatic and manual segmentation, respectively, and |⋅| denotes the cardinality of the set. For both PVD and D, the segmented labels were transformed into each participant’s native space. The average of the left and right basal ganglia PVD or D values was then computed.

## 3. Results

### 3.1. Child MRI Tolerance

Table 1 provides an overview of the MRI experience of 10 children with neuromotor conditions in an accommodative pediatric MRI suite. Though five children (50%) were able to tolerate the structural MRI scan, two of these children could not tolerate the subsequent DKI and rs-fMRI scans. In every case, it was the child’s first time having an awake MRI scan, and in every case the caregivers chose to stay in the MRI room with the child. The most common challenges reported with the MRI experience were sensitivity to scanner noise and difficulty with positioning. Four of five children who tolerated the first scan timepoint could also tolerate the subsequent timepoint (the fifth child was distressed and could not complete the second scan). During recruitment or pre-scan communications for the study, nine parents expressed hesitancy about the MRI component of the study, with only one parent confirming their child’s optional third scan timepoint. Four parents reported that their children likely did not comprehend the MRI preparation materials.

Gross motor function (items related to Lying and Sitting Dimension) (r_pb = 0.64, *p* = 0.05) and hand function (r_pb = −0.65, *p* = 0.04) were most strongly correlated with the ability to tolerate an MRI scan (Table 1). Notably, age, parent-described child motivation, and communication ability did not correlate with ability to tolerate a scan. Fisher’s exact test did not show a significant relation between CP subtype and MRI tolerance (*p* = 0.52), however the odds of tolerating a scan were lower for the participants with dyskinesia (OR = 0.25).

### 3.2. Radiologic Findings

**Figure 2:**
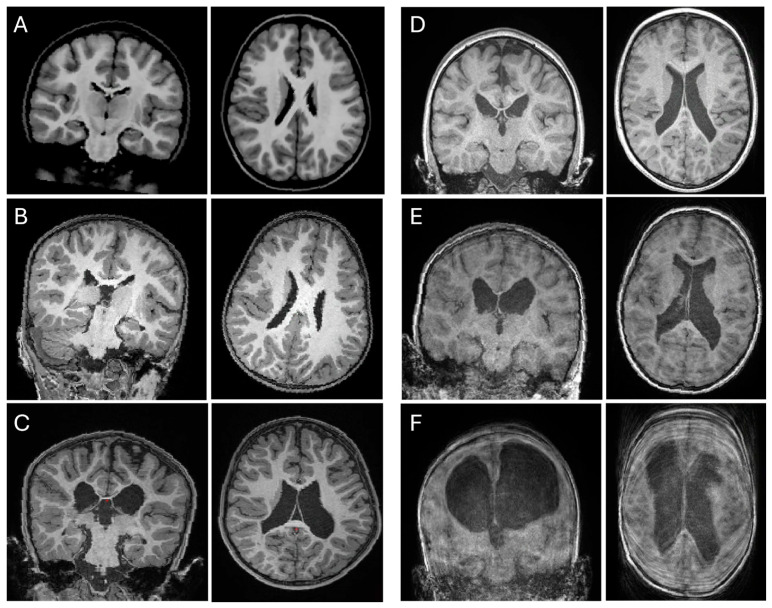
Representative coronal (**left**) and axial (**right**) T1-weighted images of *n* = 5 children that tolerated the non-sedate MRI, in order of degree of motion artifact (Child (**B**–**F**); child (**F**) with the highest degree of artifact). A neurotypical NIH template (4.5–8.5 y) is designated Child (**A**) for reference. Structural characterizations are described in Table 2.

Figure 2 demonstrates the quality and various pathologies of scans from this cohort. Scan artifacts such as ghosting and ringing are apparent in Child E and F. The NIH template (Child A) is included to show an age-matched scan that is free of motion artifact and pathology.

The radiologic findings observed in this cohort (Table 2), consistent with CP neuroimaging literature [45], were ventricular enlargement and asymmetry including periventricular leukomalacia and thinning of the corpus callosum, indicative of presumed periventricular hemorrhagic infarction (PVIH) [46]—indicative of early WM injury due to perinatal hypoxic–ischemic events [47]. Also present in scans was evidence of Wallerian degeneration of the cerebral peduncles, reflecting secondary degeneration of corticospinal tracts following childhood-onset arterial ischemic stroke [48]. One participant also showed distinctive molar tooth midbrain morphology consistent with Joubert syndrome, a neurodevelopmental disorder that can present as ataxic CP because of its cerebellar involvement. Collectively, these imaging findings align with established associations between the extent of WM injury and greater functional impairment seen in higher GMFCS levels [49].

### 3.3. MRI Quality and Cortical Surface Delineation Accuracy by Freesurfer

Though CP subtype was not significantly associated with MRI tolerance, dyskinetic participants had the lowest quality scans (Table 3). One scan had to be excluded from structural preprocessing because of significant blurring and minimal differentiation between GM and WM, making credible segmentation highly unlikely.

**Figure 3:**
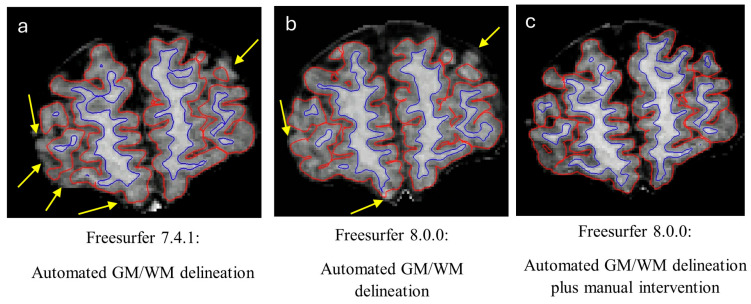
GM/WM delineation by FS, shown over cortical coronal gyri for a motion-affected participant scan (Child D; Figure 2) prior to FS deep learning integration (**a**), with FS deep learning integration (**b**), and after corrections have been applied manually following automated parcellation (**c**). Yellow arrows point to inaccuracies in the cortical surface, with the red outline delineating the pial surface and the blue outline delineating the GM/WM border.

Freesurfer’s recon-all pipeline failed for the scan rated as “Poor,” likely due to a plurality of motion-induced errors. For the scans rated as “Excellent”, the recon-all pipeline was able to complete, and the GM/WM delineations were deemed accurate by a pediatric neuroradiologist (M.S.). There were no skull stripping errors for any of the scans. For the scan rated as “Fair,” manual intervention was required following the automated pipeline to achieve accurate GM/WM delineation. Manual edits were required in the following areas: anterior temporal poles, superior gyri, and posterior occipital lobe. Pial surface inaccuracies also existed around the cortical vein and cerebellar tentorium (erroneously included in the cortex). We followed Freesurfer’s pediatric guidelines of adding more control points (placed more closely together) than recommended for adult scans. Following manual editing, the GM/WM delineation was deemed to be accurate (Figure 3).

Quantitative QA statistics showed that each pediatric scan had objectively lower CNR compared to that expected from adult scans, with the CNR decreasing with increasing motion artifact. “Defects” by the QA software in Table 3 were counted as areas affected by motion or lesions, atrophy, or cortical malformations, which accounts for their higher number than what would otherwise be expected from neurotypical brains [50].

### 3.4. Subcortical Segmentation Accuracy

**Figure 4:**
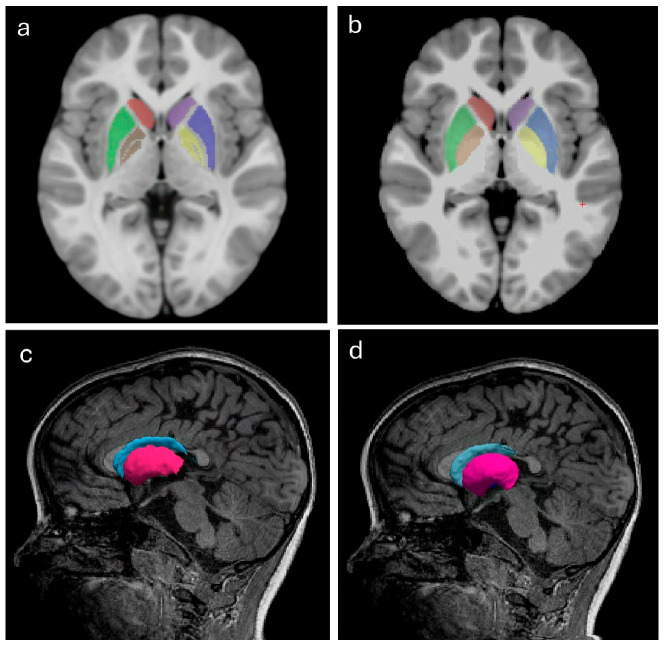
Manual segmentation of the basal ganglia using MNI Display (**a**,**c**) and automated segmentation using FS (**b**,**d**). Figures (**a**,**b**) show axial views of the caudate (red and purple), putamen (green and blue), and pallidum (brown and yellow). Figures (**c**,**d**) show sagittal views with 3D isosurface rendering of the caudate (blue) and putamen (pink).

**Figure 5:**
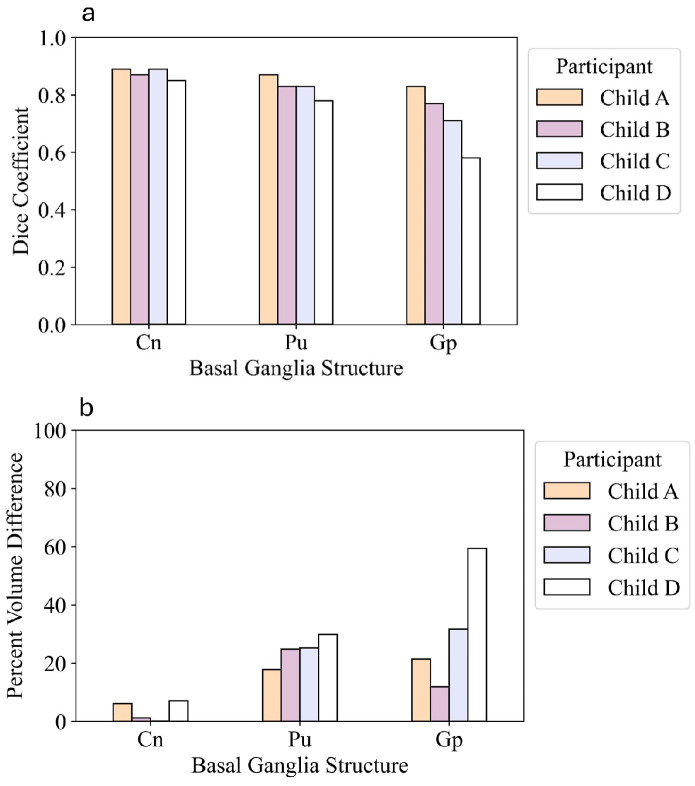
(**a**) Spatial overlap (Dice coefficient) and (**b**) percent volume difference between manual and automated segmentation methods of the basal ganglia, shown in order of degree of motion (participants corresponding to Figure 2). Caudate (Cn); Putamen (Pu); Globus pallidus (Gp).

There are visible differences between manual and automated segmentations of the basal ganglia (Figure 4). The highest spatial overlap between manual and automated subcortical segmentations was in the age-matched neurotypical NIH template (D = 0.89, 0.87, 0.83) for the caudate, putamen, and globus pallidus, respectively. This equated to volume differences of 6.1%, 17.8%, and 21.4%, respectively (Figure 5).

For the three participants and for the neurotypical NIH template, Freesurfer overestimated the volume of the basal ganglia structures compared to those derived from manual segmentation. The caudate had the lowest PVD across each participant scan (0.1–7.0%), indicating that its segmentation was least affected by motion artifacts across subjects. Volumetric differences were higher in the putamen (17.8–29.8%) and even more so in the globus pallidus (11.9–59.4%). Spatial overlap (D) between manual and automated segmentations was consistently good (D > 0.8) for the caudate, and reasonable for the putamen except in the most motion-affected scan (D < 0.8). Spatial overlap for the globus pallidus showed variability across subjects, ranging from 0.83 (good) to 0.58 (poor).

## 4. Discussion

This study examined factors related to the feasibility and tolerance of non-sedate research brain MRIs in children with CP functioning at GMFCS IV. Prior to beginning the study, most families expressed concern about the MRI component, and most declined participation in the final optional MRI scan. This hesitancy may in part reflect the high time demands of a broader mixed-methods study, of which the MRI was one component of more than 16 sessions. Lengthy and frequent study visits limited opportunities for extra preparation and desensitization activities, such as practicing with a mock scanner—known to be a successful tool for certain groups [51,52]. Additionally, parents of children with higher levels of disability already have elevated caregiving demands, elevated stress, and less time and flexibility in their daily routines [53]. Thus, experimental designs and timelines for mixed methods MRI studies should make additional provisions for pediatric participants with neurodevelopmental disabilities, allocating extra time for familiarization with procedures and behavioral desensitization, and offering family-friendly incentives for participation such as childcare provision during visits, flexible scheduling of MRI visits, and opportunities for parent and child engagement [54].

A strong predictor of non-sedate MRI feasibility is how well the child copes with other medical procedures [55]. Children with CP often require complex multidisciplinary care and face frequent invasive medical procedures which may lead to elevated medical fear and anxiety. This may further compound the fear that children in general typically have been shown to express about MRI procedures [56]. Fear of the clinical setting (MRI suite area) and fear of the magnet bore were both commonly reported barriers in our study. Interestingly, parental preparedness strongly influences MRI perception, with greater parental hesitancy related to low knowledge about MRI safety and scanning procedures [54,57]. While the majority of MRI preparation materials in our study were targeted toward child participants, it might have been helpful to provide additional materials to caregivers, since MRI literacy in the general population is low [54]. This is particularly relevant in contexts where children have limited comprehension and are wholly dependent on parental support, as was the case for several participants in our study. Cognitive impairment and communication are more prevalent in higher GMFCS levels [58], underscoring the need to prioritize caregiver education to support scan preparation and success.

In our study, age was not correlated with MRI tolerance, possibly due to the study scans being each child’s inaugural non-sedate MRI experience. Neurotypical children have self-reported difficulties with remaining still during an MRI scan [57]. This issue is amplified in children with neuromotor disorders such as CP, where there will often be involuntary, uncontrolled, and recurring movements, such as dystonia [59]. In our cohort, uncontrolled movements and difficulty with positioning were cited as barriers to scan completion. Short of using physical restraints of the head and body, managing involuntary movement during awake MRI scans is an elusive challenge. Future approaches with this population may benefit from using prospective motion correction that integrates real-time motion tracking and correction [60], retrospective motion correction utilizing k-space data [61] or specific 3D scan contrasts [62].

Of our five participants who tolerated their MRIs, only three had structural scans that were of sufficient quality for preprocessing and subsequent segmentation. In our most motion-degraded scan, manual intervention was required to correct cortical FS segmentation. Pediatric scans processed with FS tended to have consistent problem areas, particularly in the pial surface, which may fail to extend fully to the outer boundary of the cortex [18]. This underestimation can yield artificially decreased cortical thickness values, which warrants caution—particularly in populations with CP, where true cortical thinning is a documented neuroanatomical feature [63]. Nonetheless, FS’s systematic bias may be more of an issue for cross-sectional analysis, as the repeated bias may remain the same for longitudinal timepoints [18]. In circumstances when FS manual editing is required, the time allotted should be aligned with broader study objectives: manual edits may have limited impact on global measures [64], but may be more impactful when analysis is confined to small specific ROIs.

In this study, basal ganglia volumetry was used as a proof of concept for conducting pediatric volumetric analyses under motion-affected conditions. Caudate nucleus segmentation showed the highest robustness to motion in both spatial and volumetric measures, which has been noted in other literature [65]. The putamen also had clinically acceptable (D > 0.78) spatial overlap between manual and automated segmentations. As seen in our study, FS is known to overestimate subcortical volumes [66], though this bias is reportedly minimized in recent software versions. Pallidum segmentation accuracy was most dependent on scan quality and the least robust when motion was present. The size and location of the pallidum with its less clearly defined boundaries on an MRI scan probably contributed to this effect [67].

From an interpretational standpoint, accurate segmentation of brain structures in children with neuromotor impairments is essential for linking CP-related pathology to rehabilitation interventions and outcomes. Structural abnormalities, such as Wallerian degeneration, have been shown to moderately correlate with severity of contralateral sensorimotor impairments [48]. The pathology of the basal ganglia, particularly the putamen and globus pallidus, are commonly involved in dyskinetic CP [68] and is a strong predictor of gross motor impairment [69]. Understanding of these structure-function relationships contributes to frameworks such as the Magnetic Resonance Imaging Classification System for CP [22], which links structural MRI findings to rehabilitation potential, fine motor ability, and gross motor ability, and early intervention potential [22]. Combining these insights from reliable delineation of brain structures can help inform multidisciplinary care.

### Study Limitations and Future Directions

This exploratory pediatric neuroimaging study was conducted within the context of a feasibility study of exoskeleton-assisted physiotherapy with a cohort of 10 participants [26], thereby limiting its statistical power and generalizability. Although the study outcomes give a realistic picture of MRI perspectives and tolerance within a mixed methods study design, tolerance considerations may differ in a study focused exclusively on pediatric neuroimaging, since families in our study had to also attend 16 other study visits in the physiotherapy study.

Rather than including an age-matched neurotypical control or reference group, we used the NIH average age-matched neurotypical template, which may not fully capture typical developmental variability. In addition, structural MRI processing and segmentation was performed using a single software package without cross-validation with alternative methods. Subcortical segmentation was restricted to the basal ganglia, limiting the scope of investigation of anatomical segmentation accuracy.

Future work with larger sample sizes could examine feasibility when stratified across GMFCS levels, age groups, and cognitive/communication profiles to identify group-specific factors and barriers to MRI scanning and analysis pipelines. Physiological and behavioral correlates such as heart rate could also be explored as markers of stress and/or tolerance. In addition, interviews with families undergoing MRI scanning could further explore themes of scan hesitancy and medical anxiety.

Future structural analyses with pediatric neuroimaging data could benefit from parcellation and segmentation algorithms trained on pediatric data—ideally from neuroatypical populations. Incorporation of age- and pathology-specific surface and subcortical atlases would provide more anatomically appropriate references.

## 5. Conclusions

This is the first study to investigate non-sedate brain MRI tolerance and subsequent structural scan considerations for children with CP functioning at GMFCS level IV. Importantly, while our study was conducted in a resource-rich, child-centered MRI environment, several facilitators of successful non-sedated imaging, such as caregiver involvement, structured pre-scan preparation, flexible workflow, and prioritization of shorter sequence, are low-cost and broadly implementable across most clinical settings. In centers with limited resources (or geared towards adult imaging), these strategies, combined with selective referral to specialized pediatric imaging centers or use of procedural sedation when clinically indicated, may help optimize imaging feasibility and quality in children with CP.

As neuroscience and neuroimaging rely more on computational methods for automated processing, it is important to understand the limits and strengths of automated neural analysis pipelines, especially in pediatrics. The high cost of MRI scanning paired with the underrepresentation of heterogeneous pediatric populations with varying clinical severity in MRI research makes it difficult to rationalize the discarding of data. Extra considerations both to enhance the child/parent experience before and during the scanning process and to optimize subsequent structural analysis are warranted to promote inclusion of underrepresented populations in research studies.

## Figures and Tables

**Table 1:** MRI tolerance factors and challenges for children with CP (GMFCS IV).

Characteristic	Study Result
Tolerance metrics for total cohort (*n* = 10) at baseline MRI	
Children’s first time experiencing awake MRI	100% (10/10 children)
Families who expressed concern about MRI component of study	90% (9/10 children)
Families who reported children could not understand MRI prep materials	40% (4/10 caregivers)
Families who opted to stay in MRI room with their child during the scan	100% (10/10 caregivers)
Children’s structural MRI tolerance (stayed for T1W scan at minimum)	50% (5/10 children)
Children’s full MRI tolerance (stayed for T1W, DKI, and rs-fMRI scans)	30% (3/10 children)
Children who were able to complete the baseline MRI (*n* = 5)	
Children who had baseline scans repeated because of motion in MRI	60% (3/5 children)
Children who had usable quality baseline T1W for quantitative analysis	60% (3/5 children)
Children who tolerated the second MRI timepoint scan	80% (4/5 children)
Families who opted to return for a third MRI timepoint scan	20% (1/5 children)
Challenges associated with MRI experience ^1^ (as observed by research team)	
Sensitivity to internal scanner noise	4 children
Difficulty with body positioning or padding	4 children
Earbud discomfort	3 children
Difficulty with confined scanner interior	2 children
Hyperkinetic movement	2 children
Fear of clinical environment in MRI suite	2 children
Disliked audiovisual experience (tv/movie in scanner)	1 child
Participant characteristics and relation to scan tolerance	
Hand function (MACS)	r_pb = −0.65; ***p*** **= 0.04**
Gross motor function: lying and sitting domains (GMFM-88)	r_pb = 0.64; ***p*** **= 0.05**
Communication ability (CFCS)	r_pb = −0.59; *p* = 0.07
Preschool Motivation Score (DMQ)	r_pb = 0.37; *p* = 0.29
Age (range of 48–83 months)	r_pb = −0.29; *p* = 0.42
CP subtype (dyskinetic/spastic)	Odds ratio = 0.25; *p* = 0.52

^1^ Participant frequency counts may apply to more than one category. Note. T1W: T1-weighted scan; DKI: diffusion kurtosis imaging; rs-fMRI: resting state functional MRI; MACS: Manual Ability Classification System; GMFM-88: Gross Motor Function Measure, 88 item; CFCS: Communication Function Classification System; DMQ: Dimensions of Mastery Questionnaire. Statistically significant results (*p* ≤ 0.05) are indicated in bold.

**Table 2:** Participant radiologic characteristics corresponding to Figure 2.

Participant	Age at Baseline MRI	GMFCS Level	CP Classification ^1^	Clinical CP Phenotype ^1^	Radiologic Findings ^2^	Radiologic Notes ^2^: Cortical or Subcortical Involvement
Child B	4y 9m	IV	Ataxic	N/A	Molar tooth midbrain, cerebellar vermian hypoplasia	Subcortical, consistent with Joubert syndrome
Child C	5y 3m	IV	Spastic hemiparetic	Unilateral; L hemiplegia	Periventricular cystic encephalomalacia R > L, colpocephaly	Subcortical + R. Wallerian degeneration
Child D	6y 8m	IV	Spastic quadriparesis	Bilateral; UE and LE	Periventricular cystic encephalomalcia L = R, colpocephaly	Subcortical + thinning of Corpus Callosum
Child E	5y 11m	IV	Dyskinetic (dystonic)	Bilateral; UE and LE	Periventricular cystic changes L > R, colpocephaly	Subcortical + R Wallerian degeneration
Child F	4y 11m	IV	Dyskinetic (dystonic)	Bilateral (L > R); UE and LE	Marked bilateral ex vacuo dilatation with WM volume loss	Subcortical + thinning of Corpus Callosum

^1^ According to the Surveillance of Cerebral Palsy in Europe classification of CP [30] and reported by a pediatrician; ^2^ Reported by a pediatric neurologist and a pediatric neuroradiologist. Note. GMFCS: Gross Motor Function Classification System; UE: upper extremity; LE: lower extremity; L: left; R: right.

**Table 3:** Quality assessment ratings of structural T1W scans, corresponding to Figure 2, along with subsequent FS usage for structural preprocessing.

Participant	CP Subtype	Qualitative ^1^ Radiology Rating	Quantitative ^2^ Rating: WM/GM CNR	Quantitative ^2^ Rating: FS-Defined “Defects”	FS Structural Processing Usage
Child A (NT)	-	Excellent	3.01	20.5	Recon-all; no manual corrections
Child B	Ataxic	Excellent	3.17	25	Recon-all; no manual corrections
Child C	Spastic hemiparetic	Excellent	2.84	37	Recon-all; no manual corrections
Child D	Spastic quadriparesis	Fair	2.39	75.6	Recon-all; manual corrections required
Child E	Dyskinetic (dystonic)	Poor	2.07	103	Recon-all could not complete; excluded
Child F	Dyskinetic (dystonic)	Unusable	-	-	Excluded

^1^ Confirmed by a pediatric neurologist and neuroradiologist. ^2^ Derived from FS QA tool, qatools.py. Note. NT: neurotypical; WM: white matter; GM: gray matter; CNR: contrast-to-noise ratio; FS: Freesurfer; Recon-all: Freesurfer command that implements Freesurfer’s cortical reconstruction process.

## Data Availability

Data are not available on request due to privacy/ethical restrictions.

## Abbreviations

The following abbreviations are used in this manuscript:

CP	Cerebral palsy
GMFCS	Gross Motor Function Classification System
MRI	Magnetic resonance imaging
WM	White matter
GM	Gray matter
T1W	T1-weighted
T2W	T2-weighted
DKI	Diffusion kurtosis imaging
Rs-fMRI	Resting state functional MRI
FS	Freesurfer
NT	Neurotypical
GMFM	Gross Motor Function Measure
MACS	Manual Ability Classification System
CFCS	Communication Function Classification System
DQM	Dimensions of Mastery Questionnaire
CNR	Contrast-to-noise ratio
SNR	Signal-to-noise ratio
QA	Quality assessment
PVD	Percent volume difference
D	Dice coefficient
